# Potential Targeting of Renal Fibrosis in Diabetic Kidney Disease Using MicroRNAs

**DOI:** 10.3389/fphar.2020.587689

**Published:** 2020-11-13

**Authors:** Hiroko Sakuma, Shinji Hagiwara, Phillip Kantharidis, Tomohito Gohda, Yusuke Suzuki

**Affiliations:** ^1^ Department of Nephrology, Juntendo University Faculty of Medicine, Tokyo, Japan; ^2^ Department of Kidney and Hypertension, Juntendo Tokyo Koto Geriatric Medical Center, Tokyo, Japan; ^3^ Department of Diabetes, Monash University, Melbourne, VIC, Australia

**Keywords:** diabetic kidney disease, microRNA, renal fibrosis, end-stage renal disease, antifibrosis treatment

## Abstract

Diabetic kidney disease (DKD) is a major health problem and one of the leading causes of end-stage renal disease worldwide. Despite recent advances, there exists an urgent need for the development of new treatments for DKD. DKD is characterized by the excessive synthesis and deposition of extracellular matrix proteins in glomeruli and the tubulointerstitium, ultimately leading to glomerulosclerosis as well as interstitial fibrosis. Renal fibrosis is the final common pathway at the histological level leading to an end-stage renal failure. In fact, activation of the nuclear factor erythroid 2-related factor 2 pathway by bardoxolone methyl and inhibition of transforming growth factor beta signaling by pirfenidone have been assumed to be effective therapeutic targets for DKD, and various basic and clinical studies are currently ongoing. MicroRNAs (miRNAs) are endogenously produced small RNA molecules of 18–22 nucleotides in length, which act as posttranscriptional repressors of gene expression. Studies have demonstrated that several miRNAs contribute to renal fibrosis. In this review, we outline the potential of using miRNAs as an antifibrosis treatment strategy and discuss their clinical application in DKD.

## Introduction

The International Diabetes Federation reported 425 million subjects with diabetes worldwide in 2017. This number is predicted to reach 629 million by the year 2045. Diabetic kidney disease (DKD) is a major complication of diabetes and also one of the leading causes of end-stage renal disease (ESRD). Approximately 30–40% of patients with diabetes will eventually develop DKD. Although the exact mechanism underlying the development of DKD remains unknown, several causes in addition to hyperglycemia are known to contribute to its development, including genetic, environmental, and hemodynamic factors (such as hypertension, aging, arteriosclerosis, dyslipidemia, and proteinuria) (Brook, 2006; Gohda et al., 2019).

The complex pathophysiology of DKD is caused by changes in renal hemodynamics, increased oxidative stress as a result of glucose metabolic disorders, inflammatory processes, and enhanced activity of the renin-angiotensin-aldosterone system. However, the final common pathway of all these processes at the histological level is renal fibrosis, which inevitably results in ESRD.

Activated fibroblasts play a major role in the accumulation of extracellular matrix (ECM) under pathological conditions, subsequently leading to renal fibrosis. The origin of these activated fibroblasts has been extensively studied and understood to be derived from the differentiation and proliferation of resident fibroblasts, recruited from the bone marrow, and via epithelial-to-mesenchymal transition (EMT) and endothelial-to mesenchymal transition (EndMT). EMT and EndMT are the processes by which renal tubular epithelial cells and glomerular endothelial cells lose certain specific characteristics while acquiring other phenotypic properties of mesenchymal and fibroblast-like cells (Carew et al., 2012; LeBleu et al., 2013).

In recent years, activation of the nuclear factor erythroid 2-related factor 2 pathway by bardoxolone methyl and inhibition of transforming growth factor beta (TGF-β) signaling by pirfenidone have been envisioned as therapeutic targets for DKD, with a number of clinical trials being currently underway (Chin et al., 2018; Isaka, 2018).

**FIGURE 1:**
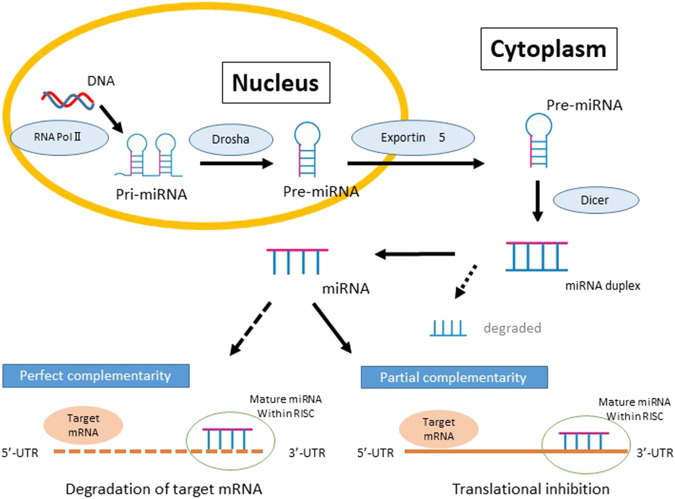
The biogenesis and function of MicroRNAs (miRNA) and the repression of gene expression. Biosynthesis of miRNAs begins in the nucleus and RNA polymerase II-dependent transcription produces capped polyadenylated transcripts known as primary miRNAs (pri-miRNAs). Pri-miRNA is processed by the RNase III endonuclease, Drosha, into a stem-loop structure known as the precursor miRNA (pre-miRNA). Pre-miRNA is transported from the nucleus to the cytosol by Exportin 5 and further processed by the second RNase III, Dicer, to generate miRNA duplexes. Posttranscriptional gene silencing occurs when the mature miRNA is then loaded into the miRNA-induced silencing complex and binds to the 3′ UTR of target mRNAs with either complete or partial complementarity.

MiRNAs, which are small noncoding RNA molecules (18–22 nucleotides), are transcribed from genomic DNA as primary miRNA (pri-miRNA) transcripts. These molecules are subsequently processed by the microprocessor complex which consists of Drosha, a nuclear RNase III, and DGCR8 (DiGeorge syndrome critical region gene 8), to yield the precursor miRNA (pre-miRNA) molecule in the form of a hairpin-loop structure (Hagiwara et al., 2013). Pre-miRNAs are then exported from the nucleus to the cytoplasm via exportin 5 where they are further processed in the cytoplasm by the ribonuclease Dicer, leading to the removal of the terminal loop to generate a mature 22-bp miRNA duplex. Finally, one of the duplex strands is loaded into the RNA-induced silencing complex (RISC) while the other strand is degraded. The RISC-miRNA complex recognizes the 3′-UTR of the target mRNA through partially complementary nucleotide sequences, ultimately resulting in the degradation of the target mRNA (Figure 1) (Knight and Bass, 2001; Lee et al., 2002; Nagalakshmi et al., 2011). In addition to 3′-UTRs, there are some miRNAs that bind to 5′-UTRs or coding regions of mRNAs and induce gene repression (Patel and Noureddine, 2012). A single miRNA can potentially modulate the expression of several genes by targeting one or more genes in various signaling pathways and therefore impact multiple biological pathways and cell function, contributing to disease (Gomez et al., 2016; Cao et al., 2019). Studies have also demonstrated the nuclear accumulation of miRNAs and roles in gene regulation by binding to promotor regions and chromatin remodeling effects (Kim et al., 2008; Place et al., 2008; Younger and Corey, 2011; Huang et al., 2012).

**FIGURE 2:**
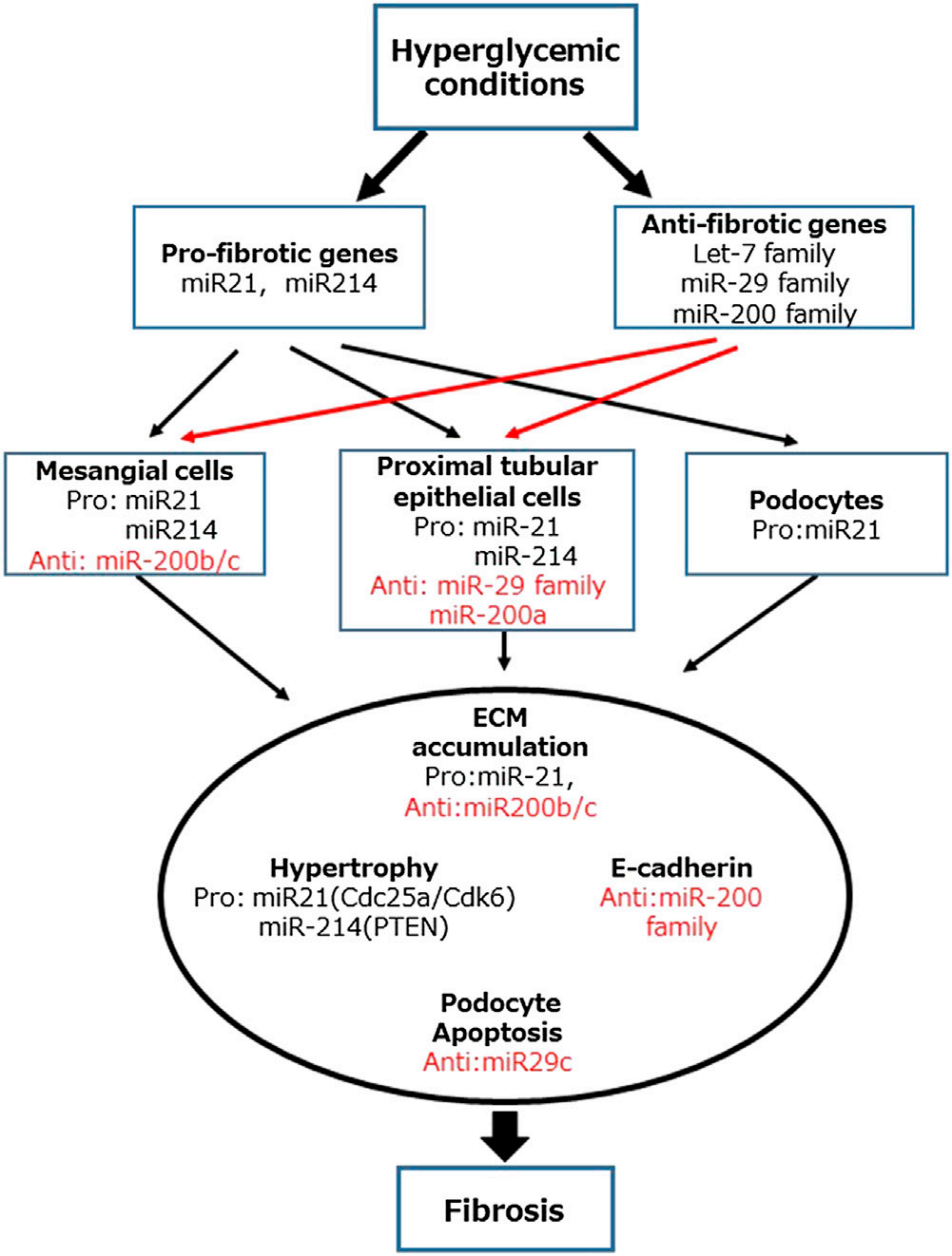
Role of MicroRNAs (miRNAs) in the development and progression of diabetic kidney disease. MiR-21 and miR-214 are classified as fibrotic genes. In contrast, let-7, miR-29, and miR-200 families are classified as antifibrotic genes.

More recently, single nucleotide polymorphisms (SNPs) in miRNAs and their connection to diabetes have also received much attention. The SNPs have been shown to impact on every aspect of miRNA biology, from transcription and biogenesis to altered targeting of miRNA to their binding sites. More specifically, some miRNA SNPs have been associated with type 1, type 2, and gestational diabetes, as well as diabetic complications (Gong et al., 2012; Li and Lei, 2015; Moszyńska et al., 2017; Zhuang and Wang, 2017; Chen et al., 2019; Zhang et al., 2019); however the impact of miRNA-related SNPs in DKD is beyond the scope of this review.

It is postulated that the interplays between metabolic and hemodynamic pathways such as hypertension, the renin-angiotensin-aldosterone system, and vasoactive hormones plays an important role in the development and progression of DKD (Cooper, 2001). We have previously reviewed the role of miRNA associated with the metabolic and hemodynamic pathways contributing to the progression of DKD (Hagiwara et al., 2013). In recent years, several miRNAs contributing to renal fibrosis and EMT have been reported and it is thought that targeting these could lead to novel antifibrotic therapeutic treatments in DKD (Reidy et al., 2014; Lin et al., 2018). We have provided a list of validated mature miRNAs and their targets relevant to DKD in Table 1. In this review, we focus on the role of miRNAs contributing to renal fibrosis in the context of DKD. Some of these miRNAs related to fibrosis are summarized in Figure 2 and are outlined below.

**TABLE 1:** Validated mature miRNAs relevant to DKD

	miRNA	Target gene
Antifibrotic	hsa-let-7b-5p	HMGA2, IGF2BP2, **TGFBR1**, JAG1, THBS1
	hsa-miR-29a-3p	**COL4A1**, **COL4A2**, **HDAC4**, LAMC2
	hsa-miR-29b-3p	SP1, **HDAC4**, TGFB1, IL6, LAMC2
	hsa-miR-200a-3p	**ZEB1**, **ZEB2**, KEAP1, TGFB2
	hsa-miR-200b-3p	**ZEB1**, **ZEB2**
	hsa-miR-200c-3p	**ZEB1**, **ZEB2**,
Profibrotic	mmu-miR-29c-3p	**Spry1**
	hsa-miR-21-5p	BCL2, **CDC25A**, PPARA, PDCD4, **PTEN**, **SMAD7**, TGFBR2, TIMP3
	hsa-miR-214-3p	**PTEN**

DKD, diabetic kidney disease; miRNAs, MicroRNAs. Source: from miRTarBase (http://mirtarbase.cuhk.edu.cn/php/index.php). Validated mRNA target genes relevant to fibrosis in DKD are shown in bold face and are discussed in this review.

### Antifibrotic MicroRNAs in Diabetic Kidney Disease

Several miRNAs associated with DKD are considered to be negative regulators of fibrotic pathways.

#### 【let-7】

Let-7, one of the first miRNAs to be discovered, was in *Caenorhabditis elegans* as an essential developmental gene (Tolonen et al., 2014). Since then, the let-7 family of miRNAs was found to be highly conserved in many species, playing a key role as inhibitory factors regulating stem cell reprogramming. This family also regulates the deposition of the ECM in breast, pancreatic, and oral cancer cells (Chang et al., 2011; Dangi-Garimella et al., 2011; Thornton et al., 2012). Moreover, the let-7 family has also been described as negative regulators of renal fibrosis. Renal let-7 expression levels were found to be decreased in a mouse unilateral ureteral obstruction (UUO) model, where upregulation of TGF-β expression is normally observed. Let-7b decreases ECM protein expression through a mechanism that involves the TGF-β mothers against decapentaplegic homolog (Smad) 3 pathway. This is probably due to the direct inhibition of let-7 on the TGF-β receptor-mediated signaling, as demonstrated in rat proximal tubular epithelial cells (NRK52E) (Brennan et al., 2013; Tolonen et al., 2014; Wang et al., 2014).

EndMT is also thought to be an important driver of renal fibrosis. The let-7 family has anti-EndMT effects, and interestingly, the fibroblast growth factor (FGF) receptor is involved in EndMT through the regulation of let-7 expression (Chang et al., 2011). The antifibrotic peptide N-acetyl-seryl-aspartyl-lysyl-proline (AcSDKP) is one of the endogenous substrates of angiotensin-converting enzyme (ACE) and hydrolyzed by it. Kanasaki et al. (Nagai et al., 2014) showed that dual treatment with ACE inhibitor (ACEi) and AcSDKP improved renal fibrosis by inhibiting EndMT more than ACEi treatment alone in diabetic CD-1 mice. The antifibrotic and anti-EndMT actions of AcSDKP have been associated with the upregulation of let-7 levels and reduced TGF-β signaling in these mice (Nagai et al., 2014; Nitta et al., 2016; Srivastava et al., 2020). Let-7 downregulated high mobility group A2 (HMGA2) which is involved in EMT in human pancreatic cancer cells. HMGA2 is a chromatin factor that is mainly expressed in undifferentiated tissues and mesenchymal tumors (Watanabe et al., 2009; Lamouille et al., 2014). Let-7 was significantly downregulated and HMGA2 was markedly upregulated in the tissue samples of DKD mice and renal mesangial cells (MCs) cultured under high glucose conditions (Wang et al., 2019). Let-7 also modulates the TGF-β pathway that is a potent driver of EMT in renal tubular epithelial cells (Wang et al., 2012a). Crosstalk between antifibrotic miRNA, in particular miR-29, and Let-7 is also important in endothelial cells homeostasis via a complex set of interactions involving FGF receptor phosphorylation and TGF-β receptor activation. This crosstalk is enhanced via the antifibrotic peptide AcSDKP, whose renoprotective action appears to be via maintenance of the cross-regulation between miR-29 and let-7 (Srivastava et al., 2019). Indeed, there is extensive crosstalk between many miRNAs and the pathways they regulate since each miRNA can target multiple genes, often in related pathways. The studying of individual miRNAs and isolated targets is often difficult because of this regulatory overlap.

#### 【miR-29】

The human miR-29 family consists of hsa-miR-29a, 29b-1, 29b-2, and 29c. MiR-29b-1 and miR-29b-2 share the identical sequence and are both referred to as miR-29b. The miR-29 family shares a common seed sequence and is generally expected to act on the same target genes. The miR-29 family has been demonstrated to exert antifibrotic effects in various organs, such as the heart and kidney (van Rooij et al., 2008; Maurer et al., 2010; Cushing et al., 2011; Roderburg et al., 2011; Xiao et al., 2012). Its other effects include the promotion of apoptosis and the regulation of cell differentiation (Kriegel et al., 2012).

Podocyte dysfunction is one of the detrimental features of DKD. The depletion of nephrin integrity may be associated with the development of diabetic podocytopathy. Lin et al. (2014) demonstrated that the levels of the podocyte injury marker desmin were increased, whereas the number of Wilms’ tumor-1-positive cells and the expression of nephrin were decreased in the glomeruli of streptozotocin- (STZ-) induced diabetic mice. Interestingly, the glomerular expression level of miR-29a, but not of miR-29b and miR-29c, was decreased in diabetic mice. When compared with diabetic wild-type mice, glomerular hyperfiltration and urinary protein levels in diabetic miR-29a-transgenic mice were significantly reduced, although blood glucose levels remained unaltered. Furthermore, miR-29a overexpression reduced nephrin loss and improved podocyte integrity probably through a mechanism involving reduction of histone deacetylase 4 levels and ubiquitination in these mice. Du et al. (2010) reported that miR-29a was downregulated by high glucose or TGF-β in human proximal tubule (HK-2) cells and that downregulated miR-29a increased the production of collagen IV protein by directly targeting the 3′UTR of *col4α1* and *col4α2*.

Renal expression of miR-29 family members was decreased with the progression of renal fibrosis in mice with UUO. However, Smad3-deficient mice with UUO were protected against renal fibrosis and increased renal miR-29 expression. Overexpression of miR-29b inhibited TGF-β-mediated induction of collagens I and III in tubular epithelial cells, whereas knockdown of miR-29b enhanced the expression of these genes, identifying miR-29b as a downstream inhibitor of TGF-β-/Smad3-mediated fibrosis (Qin et al., 2011).

Although the miR-29 family is generally considered to be protective against renal fibrosis, the data for miR-29c are discordant. Long et al. (2011) identified that Sprouty homolog 1 (Spry1), which plays a vital role in kidney development and remodeling, was targeted by miR-29c. Spry1 is considered to be a negative regulator of Rho kinase through the noncanonical Wnt signaling pathway. Several studies have reported that the inhibition of Rho kinase reduced albuminuria and mesangial matrix accumulation in experimental diabetes. High glucose downregulated Spry1 protein expression through the upregulation of miR-29c in podocytes, leading to apoptosis. Consistent with these observations, specific inhibition of miR-29c significantly reduced the high glucose-mediated induction of apoptosis in podocytes. In addition, miR-29c knockdown db/db mice exhibited decreased albuminuria through the inhibition of apoptosis, mesangial matrix accumulation, and increased fibronectin protein expression in glomeruli.

#### 【miR-200】

The miR-200 family consists of five species (-200a, -200b, -200c, -429, and -141) encoded by two separate genomic loci on chromosome 1 (Bracken et al., 2015). The mechanism through which the miR-200 family protects against renal fibrosis may involve prevention of tubular epithelial-to-EMT in proximal tubule epithelial cells (pTECs). Several studies have focused on the role of miR-200 and tubular EMT (Korpal et al., 2008; Oba et al., 2010; Wang et al., 2011; Patel and Noureddine, 2012; Xiong et al., 2012).

MiR-200a and miR-141 levels were found to be downregulated very early in the kidney of UUO mice. TGF-β mediated downregulation of the miR-200 family members is dependent on Smad signaling in pTECs. The protection against EMT by the miR-200 family is achieved by the direct targeting the zinc ﬁnger E-box-binding homeobox (ZEB) 1 and ZEB2 genes, which are transcriptional repressors of E-cadherin (Xiong et al., 2012). In contrast, the miR-200 family was upregulated in the UUO model, with the induction of miR-200b being the most pronounced. Intravenous administration of miR-200b precursor improved renal fibrosis in UUO and increased the expression of both ZEB-1 and ZEB-2 (Oba et al., 2010).

### Profibrotic MiRNA in Diabetic Kidney Disease

#### 【miR-21】

MiR-21 has been widely investigated because several of its targets that are relevant to DKD and especially related to TGF-β were found to induce the activation of phosphoinositide 3-kinase- (PI3K-) AKT signaling (Godwin et al., 2010; Zhong et al., 2011). Moreover, it has been reported that TGF-β upregulated miR-21 expression in the liver, heart, lung, and kidney in mice and was involved in TGF-β-induced fibrosis in these tissues (Zavadil et al., 2007; Davis et al., 2008; Zhong et al., 2011; Loboda et al., 2016). TGF-β stimulation upregulated the expression of miR-21 in pTECs. Interestingly, Smad3, but not Smad2, was involved in the induction of miR-21 in response to TGF-β. Furthermore, mice deficient in Smad3 were found to be protected against the upregulation of miR-21 and renal fibrosis in the UUO model. Indeed, miR-21 expression and renal fibrosis were promoted in Smad2-knockout UUO mice. Gene transfer of a miR-21-knockdown plasmid was found to cease the progression of renal fibrosis in the UUO model. These results demonstrated that Smad3 signaling promoted the expression of miR-21 in the UUO mice (Zhong et al., 2011).

Dey et al. (2012) showed that phosphatase and tensin homolog (PTEN) acts as a target gene of miR-21 in human glomerular MCs. Upregulation of miR-21 by TGF-β stimulation downregulated the expression of PTEN, resulting in the activation of AKT and mammalian target of rapamycin complex 1, which regulated MC hypertrophy (Mahimainathan et al., 2006; Kato et al., 2009; Dey et al., 2012).

McClelland et al. (2015) reported that upregulation of miR-21 in the kidney was positively associated with the severity of fibrosis and renal dysfunction in patients with DKD. Using rat pTECs, they demonstrated that TGF-β promoted renal fibrosis by inducing miR-21 which in turn targets Smad7 and PTEN, the negative regulators of Smad3 and PI3K, respectively.

In diabetic KK-*A* ^*y*^ mice, the expression of miR-21 was observed predominantly in cortical glomerular and renal proximal tubular cells. The expression of miR-21 was positively correlated with the urine albumin–creatinine ratio, as well as TIMP1, collagen IV, and fibronectin protein levels, and negatively correlated with the creatinine clearance ratio and MMP-9 protein levels (Wang et al., 2013).

Cell division cycle 25a (Cdc25a) and cyclin-dependent kinase 6 (Cdk6) were identified as targets of miR-21 in mouse MCs. MiR-21 directed the inhibition of Cdc25a and Cdk6 and led to MC hypertrophy via a mechanism that impaired cell cycle progression. Furthermore, miR-21 antagonism in a STZ-induced diabetic mouse model resulted in reduced fibrotic and inflammatory gene expression, as well as reduced mesangial expansion, podocyte loss, interstitial fibrosis, macrophage infiltration, and proteinuria (Kolling et al., 2017).

Liu et al. (2019) reported that bone morphogenetic protein 7 (BMP-7), a human recombinant protein, inhibited EMT and ECM synthesis and accumulation in rat renal tubular epithelial (NRK-52E) cells cultured under high glucose conditions. Moreover, injection of a BMP-7-overexpressing plasmid to STZ-diabetic mice caused a significant decrease in miR-21 expression and upregulated Smad7 expression, thereby leading to the prevention of EMT and ECM accumulation. These data support the view that the protective effect of BMP-7 against renal fibrosis in DKD is in part via regulation of miR-21 and Smad7 signaling.

The bioactive saponin Astragaloside IV (AS-IV), which is extracted from astragalus root, is known to have therapeutic effects on conditions such as liver fibrosis, DKD, and chronic medical heart failure (Gui et al., 2006; Wang et al., 2012b; Guo et al., 2017). Wang et al. demonstrated that AS-IV decreased the expression of miR-21 in cultured mouse MCs, mouse primary podocytes, and serum and kidney of diabetic KK-*A* ^*y*^ mouse. In MCs and podocytes, overexpression of miR-21 enhanced signaling via the TGF-β/Smad and the β-catenin signaling pathways, which was abolished by AS-IV treatment. It was reported that AS-IV improved renal function and fibrosis by a mechanism that involved prevented increased miR-21 expression and thereby preventing podocyte dedifferentiation and MC activation in mice with DKD (Wang et al., 2018).

#### 【miR-214】

High expression levels of miR-214 have been detected in human and animal models of kidney disease (Gomez et al., 2016). MiR-214 is cotranscribed with miR-199a as a single long noncoding RNA from an intron on the complementary strand of the dynamin-3 gene. The upregulation of both miR-214 and miR-199a is driven by the TWIST transcription factor and HIF-1-mediated hypoxia (Lee et al., 2009; el Azzouzi et al., 2013; Chen et al., 2014).

The antifibrotic effect was observed when the anti-miR-214 drug was administered to mice before the induction of UUO. In the UUO model, inhibition of canonical TGF-β signaling did not change endogenous miR-214 expression but blocked Smad2/3 activation. In contrast, treatment with miR-214 antagonist in mice did not prevent the activation of Smad2/3. Moreover, TGF-β inhibition when combined with deletion of miR-214 resulted is superior renal protection than miR-214 deletion alone. It was demonstrated that miR-214 has a fibrotic effect independent of Smad2/Smad3 activation (Denby et al., 2014).

Gene profiling revealed a significant upregulation of renal cortical miR-214 expression in diabetic db/db mice. In human embryonic kidney cells 293, PTEN was identified as a target of miR-214. Inhibition of miR-214 was observed to significantly decrease the expression of collagen IV, α-SMA, and SM22. In the same study, miR-214 inhibition also partially restored PTEN protein levels in human MCs under high glucose conditions as well as in db/db mice. Furthermore, this inhibition attenuated albuminuria and mesangial expansion in diabetic mice. Moreover, overexpression of PTEN was found to ameliorate MC hypertrophy, whereas knockdown of PTEN promoted MC hypertrophy (Wang et al., 2016).

#### 【miR-199a】

As previously mentioned, miR-199a is cotranscribed with miR-214. While several studies have investigated miR-199a and its relevance to tissue fibrosis, the role of miR-199a in kidney disease and particularly in DKD has not yet been established.

The expression of miR-199a-5p was found to be increased in TGF-β-stimulated lung fibroblasts, UUO mice, and mice with CCl4-induced liver fibrosis, suggesting that dysregulation of miR-199a-5p contributes to the fibrogenesis. *In vitro* studies have demonstrated that miR-199a-5p is a key downstream mediator of TGF-β signaling in lung fibroblasts where it targets caveolin-1, an important mediator of pulmonary fibrosis (Lino Cardenas et al., 2013).

Sun et al. (2015) demonstrated that miR-199a-5p expression was dramatically increased in the renal tissue of patients with autosomal dominant polycystic kidney disease (ADPKD), in the renal tissue of the rat ADPKD model, and in human ADPKD in the epithelial cell lining. The target gene involved here was found to be cyclin-dependent kinase inhibitor 1C (CDKN1C)/p57. Increased expression of miR-199a in the ADPKD renal tissue may promote cell proliferation through the suppression of CDKN1C.

### Therapeutic Strategies for Diabetic Kidney Disease Using MicroRNAs

Dysregulation of TGF-β by resident renal cells and infiltrating inflammatory cells that are subject to stress in response to high glucose, angiotensin II, and reactive oxygen species, is a key factor contributing to renal fibrosis. TGF-β causes MC hypertrophy and proliferation, the induction of podocyte apoptosis and detachment from the glomerular basement membrane, ECM synthesis and accumulation, and other structural and functional changes in the kidney (Wu and Derynck, 2009; Boor and Floege, 2011; Rask-Madsen and King, 2013; Meng et al., 2016; Ma et al., 2019). Drugs targeting signal transduction pathways such as TGF-β have been developed for the treatment of DKD with limited success due to the important functions these pathways exert in normal physiology. As detailed earlier, several miRNAs have been implicated in the development and progression of DKD. Recent efforts were focused on applying the current knowledge regarding miRNA structure and function to develop novel miRNA therapeutics for DKD. Novel strategies were focused on inhibiting those miRNAs that are induced by DKD or increasing the expression of renoprotective miRNA (Lennox and Behlke, 2011; Trionfini et al., 2015; Lima et al., 2018).

MiRNA mimics for therapeutic use are designed to mimic the endogenous miRNA. They are double-stranded synthetic oligonucleotides that are processed in cells to mimic the endogenous function of miRNA, with improved stability and chemical modifications that enable efficient delivery and entry into target cells. The inhibition of endogenous miRNA may be achieved by introducing anti-miRNA oligonucleotides that target pri-miRNA, pre-miRNA, or mature miRNA to sequester or remove endogenous miRNA (Weiler et al., 2006; Kato et al., 2016). Although miRNAs are generally considered to be stable, individual miRNAs can rapidly decay in certain cellular environments (Trionfini et al., 2015). Several modifications have been made to increase RNA stability *in vivo*, which include 1) replacing the phosphodiester backbone with a phosphorothioate backbone, 2) ribose 2′-OH group, 3) locked nucleic acid modifications, and 4) peptide nucleic acid modification (Lennox and Behlke, 2011; Cao et al., 2019).

MiRNA may be a novel and attractive target for the treatment of DKD; however, several obstacles must be overcome to move miRNA-based therapies into clinical trials. Targeting miRNAs to the kidney remains a significant challenge in order to avoid potential unwanted effects in other tissues and organs, as well as off-target effects. Using miRNA mimics or inhibitors *in vivo* is considered to be a promising therapeutic strategy for the treatment of DKD. In fact, successful delivery of mimics and inhibitors to the kidney has been achieved via intravenous and subcutaneous injections (Trionfini et al., 2015).

Miravirsen, an anti-miR-122, is the first drug targeted for miRNA, and a phase II trial in patients with hepatitis C virus infection has been conducted. The use of Miravirsen in patients with chronic HCV genotype 1 infection prolonged reduction of HCV RNA levels (van der Ree et al., 2016). With further developments in this area, it is envisaged that targeting various miRNAs would be introduced to clinical practice as a nephroprotective treatment approach in the future.

## Conclusion

DKD is a major complication of diabetes and a leading cause of ESRD. It is a complex multifactorial disease, which involves several physiological pathways leading to fibrosis. In recent years, various therapeutic agents targeting fibrosis have been investigated for DKD treatment, and some clinical trials have been conducted; however, no useful therapeutic agent has been found till date. MiRNA profiling may provide a better understanding of the complex pathways of DKD progression, and inhibition or overexpression of miRNA may lead to miRNA-based therapeutics in the future.

## Author Contributions

HS wrote and edited the manuscript. SH drafted and wrote and edited the manuscript. PK edited and revised the manuscript. TG reviewed and edited and revised the manuscript. YS reviewed and edited the manuscript.

## Funding

This manuscript was supported by JSPS KAKENHI Grant Number 18K08220 and the NHMRC (#225940, #1183737).

## Conflict of Interest

The authors declare that the research was conducted in the absence of any commercial or financial relationships that could be construed as a potential conflict of interest.
